# Surgery priority at the time of COVID-19 pandemic, a conceptual frame work recommendation

**DOI:** 10.22088/cjim.11.0.577

**Published:** 2020

**Authors:** Atieh Akbari, Narjes Mehrvar, Mohammad Esmaeil Akbari

**Affiliations:** 1 Cancer Research Center, Shahid Beheshti University of Medical Sciences, Tehran, Iran; 2 MAHAK Hematology Oncology Research Center (MAHAK-HORC), MAHAK hospital, Shahid Beheshti University of Medical Sciences, Tehran, Iran

**Dear Sir,**

The current pandemic disease, COVID-19 is causing fear and affecting the quality of life in 2020. The mentioned disease can spread human to human, especially by asymptomatic carriers. Healthcare providers and surgeons are at the risk of conferring with COVID-19. Surgical history in pandemic situations is rare.

 In this regard, the present study designed suggesting practical and fundamental issues around the efficacy of surgery at the time of COVID-19 pandemic. This information and recommendations could be as a valuable source for surgeons in low and middle-income countries. In December 2019, the novel Severe Acute Respiratory Syndrome Coronavirus 2 (SARS-COV2) from the coronaviridea family occurred in Wuhan, Hubei Province, China (1). Surfaces are an important route of transmission for this pathogen. Viruses can contaminate the inanimate environments of every place including the hospitals’ surfaces (2). The stability of SARS-COV viruses on inanimate environments depends on the type of the strain, the surface, temperature and humidity (3). Accordingly, hospital-related transmission could definitely be an important and challenging issue (4). 

Operating rooms (OR) could be considered as one of the main contaminated wards in hospitals because of staff traffic and behaviors; in addition, due to the airborne particles and persistence of the particles on surfaces within a closed air circulation (5). For this reason, patients who are confronted by the virus during or after the surgery will have a higher mortality rate than non-hospitalized patients (6). 

Due to the mentioned issues and the current situation of COVID-19, making decisions about elective surgeries is as important as saving humans’ lives. Herein, we offer a conceptual recommendation for evaluating the necessity of performing elective surgeries in health care centers. 

**Surgery in pandemic situations: **Surgery has a main role in healthcare systems and is the procedure of choice for treatment of patients with different diseases, especially in low- and middle-income countries. During a pandemic situation, healthcare providers and the surgery team are greatly concerned about the risk of confronting the disease. In this regard, prioritizing resources at the time of crisis is the most important issue. Due to the epidemiological pattern of COVID-19, false negative tests, transmission from asymptomatic cases, permanency of the virus in the operating rooms from previous surgeries and circulation of the virus within the community, and the necessity of elective surgeries for many cases, we divided the effective criteria into three statuses:

1-criteria belong to patient which is related to disease and outcome of that by performing or not performing surgery during pandemic. Iranian official guidelines from the Ministry Of Health and Medical Education (MOH&ME) on elective surgery is categorized into necessary, semi necessary and unnecessary, with regard to this classification you have to postpone surgery for one to twelve months to prepare suitable condition (7). 

2-criteria belong to place of surgery: With Iranian guidelines, elective surgery in a center which is responsible to care of covid-19 cases is completely prohibited, NHS recommends home delivery oral medication rather than visit and intravenous administration. ESMO and ESSO recommend no visiting cases aged more than 70 years old and reducing clinical visit by replacing telemedicine. These are because of more probability in infected health centers and hospitals. The intra hospital transmission is more common rather than other public places, virus will persist in the hospital surfaces specially in the operating theaters (OR) because of more soft surfaces such as steel and stainless steel and appropriate humidity and temperature (8). 

3- Criteria belong to surgical team: They have to be sure about their safety from covid-19, with minimal number in the operating room, decision must be taken in multi-disciplinary team (MDT), be well aware about the hospital and OR facilities, the surgeon and his/her team must judge well the patient’s condition based on up to date knowledge and institutional or national guidelines which are completely related to extent of covid -19 infection (9). Finally our recommendations for surgery during pandemic of corona virus are as follows:

- Surgery is mandatory for all urgent cases and life threatening conditions, with protective measures
- Surgeries should be omitted as much as possible even in an emergency status. Many cases should be managed by non-operative management and less hospitalization, such as vaginal delivery, bone fractures, inflammatory processes like the early phase of appendicitis and cholecystitis. Decision for this condition are made by the surgeon and his/her multi-disciplinary team.
- Surgery for improving survival rates should be classified and prioritized scientifically, more survival expectation and cure probability have more priority.
- Surgery is not recommended for improving the quality of life of individuals, for example all cosmetic and reconstructive surgeries until the condition of epidemic and hospital status is acceptable.
- The surgery team should pay attention to the safety of laparoscopic and open surgeries. However, laparoscopic procedures have a high risk of aerosol formation and infection.

In summary, surgery with all protective measures is strongly recommended for life threatening situations during an epidemic. Otherwise, it should be given more consideration for the cases which are not urgent or no beneficence for survivorship and quality of life depends on up to date knowledge, guidelines, available facilities and healthy places.
